# Reward Value Enhances Sequence Monitoring Ramping Dynamics as Ending Rewards Approach in the Rostrolateral Prefrontal Cortex

**DOI:** 10.1523/ENEURO.0003-22.2022

**Published:** 2022-03-04

**Authors:** Theresa H. McKim, Theresa M. Desrochers

**Affiliations:** 1 Department of Neuroscience, Brown University, Providence, RI 02912; 2 Department of Psychiatry and Human Behavior, Brown University, Providence, RI 02912; 3 Robert J. and Nancy D. Carney Institute for Brain Science, Brown University, Providence, RI 02912

**Keywords:** cognitive sequence, executive control, fMRI, prefrontal cortex, reward

## Abstract

Many fundamental human behaviors contain multiple sequences performed to reach a desired outcome, such as cooking. Reward is inherently associated with sequence completion and has been shown to generally enhance cognitive control. However, the impact of reward on cognitive sequence processing remains unexplored. To address this key question, we focused on the rostrolateral prefrontal cortex (RLPFC). This area is necessary and exhibits increasing (“ramping”) activation during sequences, a dynamic that may be related to reward processing in other brain regions. To separate these dynamics, we designed a task where reward was only provided after multiple four-item sequences (“iterations”), rather than each individual sequence. Using fMRI in humans, we investigated three possible interactions of reward and sequential control signals in RLPFC: (1) with the visibility of sequential cues, i.e., memory; (2) equally across individual sequence iterations; and (3) differently across individual sequence iterations (e.g., increasing as reward approaches). Evidence from previous, nonsequential cognitive control experiments suggested that reward would uniformly change RLPFC activity across iterations and may depend on the visibility of cues. However, we found the influence of reward on RLPFC ramping increased across sequence iterations and did not interact with memory. These results suggest an active, predictive, and distinctive role for RLPFC in sequence monitoring and integration of reward information, consistent with extant literature demonstrating similar accelerating reward-related dopamine dynamics in regions connected to RLPFC. These results have implications for understanding sequential behavior in daily life, and when they go awry in disorders such as addiction.

## Significance Statement

We daily perform multiple sequences to achieve rewarding goals, but little is known about how control of these sequences and reward information interacts in the brain. The rostrolateral prefrontal cortex (RLPFC) is necessary to perform sequential tasks and contains reward-related signals, making it a key potential site of integration. We designed a human fMRI experiment to test three possibilities for how reward and sequential control processes may interact: (1) through a nonsequential process; (2) uniformly across sequences; (3) nonuniformly across sequences. We found that reward shows greater interaction with sequential control dynamics in the RLPFC as the ending reward approaches. These results provide insight into the function of RLPFC and how rewards are integrated into temporally extended control processes.

## Introduction

A hallmark of goal-directed behavior in everyday life is the performance of sequential tasks to reach a reward. For example, when cooking, a series of tasks, such as chopping, adding ingredients, and stirring, must be monitored and executed before sitting down to a satisfying meal. Completing these tasks requires cognitive control over items in the progression (“sequential control”). Progress has been made in understanding how rewards generally enhance nonsequential cognitive control behaviors and neural dynamics (Braver et al., 2014; Botvinick and Braver, 2015; Yee and Braver, 2018; Chiew, 2021), but how rewards may modulate sequential control processes remains an open question.

Sequential control and reward both underlie dynamics in the rostrolateral prefrontal cortex (RLPFC; also referred to as anterior prefrontal cortex or lateral frontal polar cortex). Human functional magnetic resonance imaging (fMRI) and transcranial magnetic stimulation (TMS) experiments have shown that RLPFC is necessary for sequential control and monitoring (Desrochers et al., 2015, 2019). In nonsequential tasks, RLPFC tracks reward trends (Kovach et al., 2012), demonstrates increased task-related activity with increased reward (Pochon et al., 2002), and correlates with individual differences in reward activation (Locke and Braver, 2008). Thus, RLPFC is an ideal initial focus for examining dynamics in a network integrating reward information with sequential control.

The RLPFC also shares key connections and similarities in dynamics with regions and reward-related neurotransmitters. In this area, activity increases (“ramps”) from the first to the last position of the sequence (Desrochers et al., 2015, 2019). Other reward-related brain regions such as the anterior cingulate cortex (ACC), striatum, and ventral tegmental area (VTA) show ramping neural activity as rewards approach (Totah et al., 2013; Ma et al., 2014; Wang et al., 2018) and dopamine concentration increases (Howe et al., 2013; Hamid et al., 2016). RLPFC is anatomically connected to these (Desrochers and Badre, 2012; Haber and Behrens, 2014; Coenen et al., 2018) and other reward processing regions such as the orbitofrontal cortex (Thiebaut de Schotten et al., 2012) and ventromedial prefrontal cortex (Haber and Behrens, 2014). Similar dynamics in RLPFC and connected reward-related regions further raises the potential for interaction of processing-related activity within RLPFC.

Typically, the influence of reward is studied in association with a single action or event. In the less frequent situation where reward is studied in conjunction with sequences, it often occurs at the end of a single sequence, making the effects of reward and sequence completion inseparable. Therefore, to disentangle the effects of reward and sequence on RLPFC activity we designed a task, described in further detail below, that required participants to complete multiple four-item sequences before receiving reward. We term a single instance of a four-item sequence as an “iteration.” Participants performed several iterations of the same sequence (e.g., cutting up multiple items while cooking) to obtain reward. With this paradigm, we examined the interaction between reward and sequence processing signals in the RLPFC.

Reward and sequential control dynamics could interact in several ways across multiple sequence iterations. First, reward effects could be uniform but nonspecific to sequence iterations. For example, several memory processes influenced by reward have been localized to RLPFC (Shallice et al., 1994; Christoff et al., 2001; Braver and Bongiolatti, 2002; Shigemune et al., 2017). Second, reward may have a uniform influence across items within individual sequences. In nonsequential tasks, RLPFC exhibits tonic (Beck et al., 2010) and increasing (Chiew et al., 2016) reward-related activity changes. Third, reward may have an influence that is nonuniform across sequence iterations (e.g., increasing as reward approaches). Serial behaviors (Braun et al., 2018) and computational modeling (Ott et al., 2020) suggest that reward information representation changes as temporally distant rewards approach. Further, dopamine concentrations in the striatum show accelerating changes as reward approaches (Howe et al., 2013), but only in regions directly involved in task completion (Hamid et al., 2021). Thus, the nature and distribution of potential interactions has consequences for how RLPFC controls sequential processes in the presence of reward.

To test these alternatives, human participants monitored multiple sequence iterations under differing reward and cue visibility conditions to manipulate memory demand. Based on reward influence on nonsequential cognitive control (Beck et al., 2010; Chiew et al., 2016), we hypothesized that reward would change RLPFC sequence monitoring dynamics uniformly across individual sequences. We found that reward did not change the slope or magnitude of individual sequence ramping or interact with memory demand in RLPFC. Instead, we provide novel evidence that reward interacts with sequential dynamics only as the end reward approaches. These results suggest that nonlinear increases in activity proximal to reward outcome realization is important for assigning value to temporarily extended cognitive control processes, and further implicate RLPFC as a crucial node in processing abstract sequence information.

## Materials and Methods

### Participants

Thirty-six (*n* = 20 female) adults between the ages of 18–35 years of age [M (mean) = 24; SD (standard deviation) = 5] were included for analysis in the experiment. An additional 18 participants performed the task but were excluded from analyses because of poor participant performance, poor data quality, and equipment malfunction issues and are detailed as follows. Five participants were unable to complete the task and/or fell asleep. One participant had a previously undetected brain abnormality and was subsequently excluded from analysis. One participant had very low signal-to-noise ratio (SNR), and four participants had excessive head motion (>3 mm). *Post hoc* it was discovered that seven participants completed the task when the response button box began malfunctioning (MR compatible four button response pad, Mag Design and Engineering). This malfunction resulted in an inability to dissociate participant error from response box malfunction and a large number of trials that had to be excluded for incorrect responses (>30%). Therefore, we excluded these seven participants and the button box was replaced with a fiber optic response pad (five button handheld device, Cambridge Research Systems) for the remaining participants. We confirmed there were no differences in reaction times (RTs) between participants based on the response device for all behavioral analyses reported below (all *F*s <3.92, *p*s > 0.07).

All participants were right-handed, had normal or corrected-to-normal vision, and reported they were not colorblind. Individuals with neurologic or psychiatric conditions, brain injury, or reported use of psychoactive medications or substances were excluded from participating. Participants were recruited from the Brown University campus and the surrounding community. Participants were compensated $20/h for their time. Additionally, participants received up to a $10 bonus based on task performance. Specifically, participants were instructed before scanning that one run would be randomly selected after task completion to determine the performance bonus. This instruction was used to motivate and encourage task performance across all scanning runs. In reality, all participants received a $10 bonus for task completion. All participants gave informed, written consent as approved by the Human Research Protections Office at Brown University.

### Task design and procedure

#### Overview

**Figure 1.**
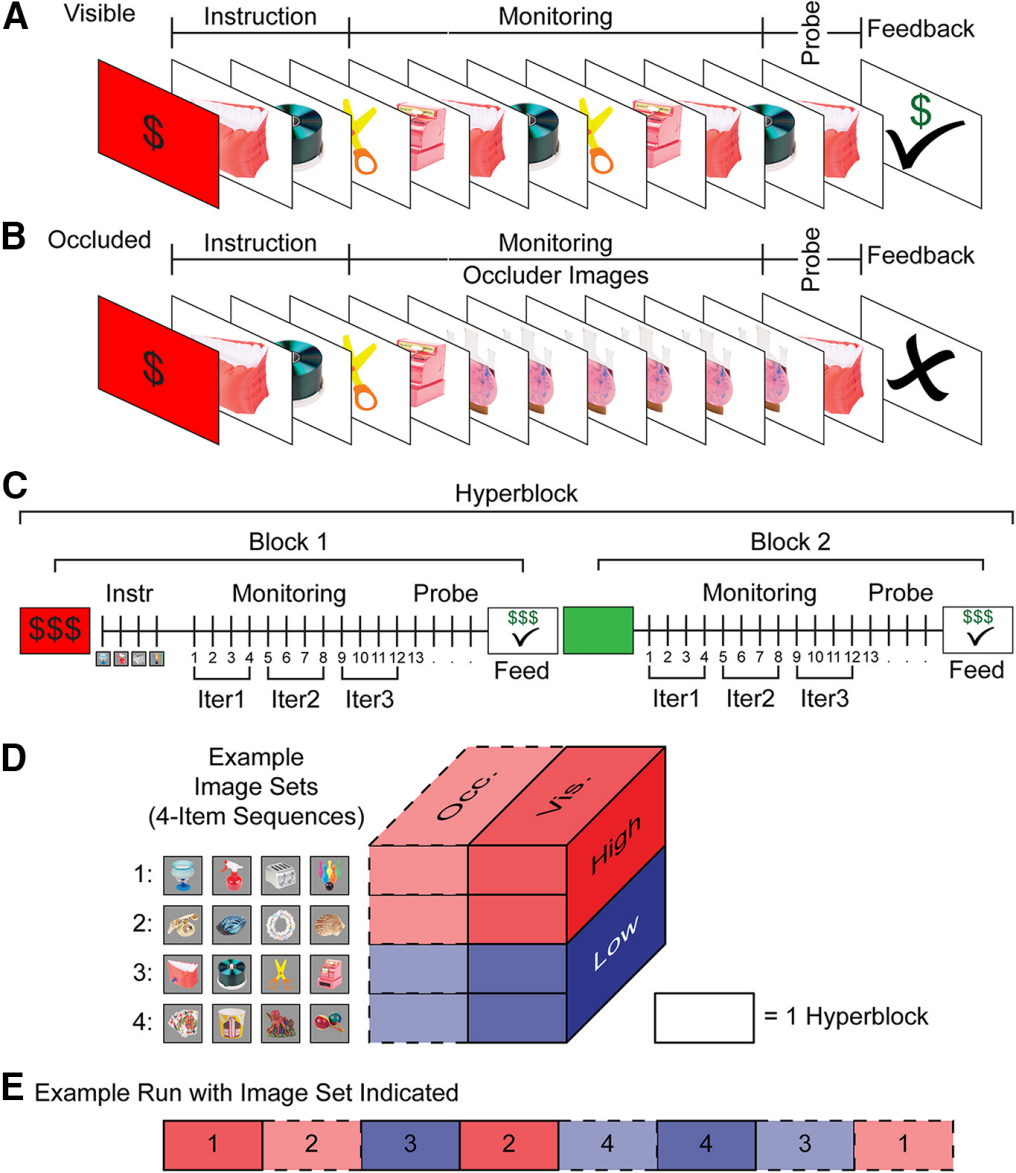
Sequence monitoring task with reward. Across all conditions, the first block starts with a red instruction screen (reward indicated by amount of dollar signs, $$$ or $). Instruction images (Instr), that participants do not respond to, are serially presented to show the correct order of the current image set. Participants then respond on each monitoring trial (1 s) with an in or out of sequence button press. After the last image (probe) of the block, feedback (Feed) is given for that trial in the form of a check mark (correct; dollar signs above to indicate reward) or an X for an incorrect response. ***A***, Example Visible condition block that is Low value (indicated by one $) and has correct feedback (check mark and $). The instructed image set stimuli are visible across all monitoring trials. ***B***, Example Occluded condition (Low value, same image set as in ***A***) and has error feedback (X). The “occluder” image is displayed for monitoring trials after instruction. Participants monitor the stimuli as if the images from the sequence are occluded by this placeholder image. Only the probe image is a member of the instructed image set. ***C***, Hyperblock structure. A “hyperblock” is two consecutive blocks of the same condition (High value, indicated by $$$, illustrated here), created to maximize the number of monitoring trials (by eliminating the instruction images from the second block). The second block begins with a green screen to indicate the continuation of the same condition. Individual blocks contain multiple sequence “iterations” (Iter), defined as one four-item sequence or ordered image set. All iterations within the same block are from the same image set. ***D***, Schematic of experimental conditions. Four image sets are randomly chosen for each participant. Each image set is only associated with either High or Low value and both Occluded and Visible conditions. ***E***, Example run structure. Each run contains eight hyperblocks (16 blocks), one of each condition by image set combination such that there are two hyperblocks for each of the four conditions (Visible/Occluded × High/Low). Numbers in the schematic indicate the image set as in ***D***. The order of hyperblocks was counterbalanced within and across runs. Vis = Visible condition. Occ = Occluded condition. Solid lines with darker shades indicate the visible condition. Dashed lines with lighter shades indicate the Occluded condition. Red tints and shades are High value. Blue tints and shades are Low value.

Participants completed a modified version of the sequence monitoring task published in Desrochers et al. (2019). Participants monitored serially presented four-item sequences and for each item, indicated whether it was in or out of the preinstructed order with a button press (Fig. 1). Each participant completed a single session that included task training, a short sequence preference test, task performance while undergoing fMRI scanning, and post-scanning sequence preference test and questionnaires.

#### Trial structure

Sequences were composed of four unique visual stimuli drawn from a pool of common objects (Desrochers et al., 2019). Four image sets were drawn randomly for each participant. Task stimuli were displayed using an Apple computer running macOS. Experiment scripts were programmed using the Psychophysics Toolbox in MATLAB (MathWorks; RRID:SCR_001622). On each trial, a stimulus was displayed in the center of the screen on a gray background. Participants were asked to press a button to indicate whether the displayed image was in or out of the instructed sequence order while the image was presented on the screen for 1 s. An intertrial interval (ITI) followed each image and displayed a fixation cross centrally. The ITI timing was jittered and optimized across trials for scanning (0.25–8 s, mean 2 s).

Participants responded with the index and middle fingers on their right hand. One key was assigned the “in sequence” response and the other was the “out of sequence” response. Response options were counterbalanced across participants. Participants were instructed to respond as quickly and accurately as possible while the stimulus was displayed on the screen (1 s). Occasional responses (mean 8%, across participants) that occurred in the ITI were included in analyses to avoid unnecessary data loss.

#### Block structure

Rewards were infrequent in the task structure because participants completed multiple four-item sequences before obtaining reward. Therefore, we adopted a nested block structure to maximize the number of sequence and reward trials within a reasonable amount of time for the participant to perform the task in the scanner (∼ 1 h). This nested structure saved time by allowing two blocks to be completed with only one instruction period. An overview of the terminology and structure follows:

- Condition: one of the four possible combinations of the two-reward by two-visibility type design: Visible High, Visible Low, Occluded High, Occluded Low (Fig. 1*A–D*).
- Image set: one of four prelearned sets of image orders (see below, Training). Each image set was only associated with either High or Low value. However, each image set was used for both Occluded and Visible trials (Fig. 1*D*).
- Hyperblock: two, consecutive blocks of the same condition (Fig. 1*C*). Consecutive hyperblocks would not be the same condition, as the order was randomized (Fig. 1*E*).
- Block: a group of monitoring trials, probe, and reward (Fig. 1*A–C*).
- Red screen: signal to get ready at the beginning of the first block of a hyperblock, and that a new condition was starting (Fig. 1*A–C*).
- Instruction images: only occurred in the first block of a hyperblock after the red screen. The four images from one image set were displayed, in order, for 0.75 s each with no time between images. The participant did not respond to these images (Fig. 1*A–C*).
- Green screen: signal to get ready at the beginning of the second block of a hyperblock. Because it was the same condition as the first block, no value cue was shown, no instruction images followed it, and participants pressed the button to proceed immediately to monitoring trials (Fig. 1*A–C*).
- Monitoring trials: participants responded to indicate whether each image was in or out of sequence (as described above, Trial Structure) for a total of 13–16 consecutive trials (Fig. 1*A–C*). In the Visible condition, these images were all from the image set, and in the Occluded condition, all images were the placeholder image. Monitoring trials can be further grouped into:

∘  Sequence iterations: one set of four items that made up the instructed sequence (Fig. 1*C*). All iterations within a block were from the same image set.

∘  Iteration 1: trials 1–4 were not different from subsequent monitoring trials for the participant, but we excluded them from behavior and fMRI analyses to avoid block initiation effects.

∘  Iteration 2: trials 5–8. The first iteration used in analysis.

∘  Iteration 3: trials 9–12. The second iteration used in analysis.

- Probe trial: the probe trial occurred, with equal probability, during the fourth iteration on trial 13, 14, 15, or 16. In other words, the probe trial occurred with equal probability across the four positions (1–4) in the sequence iteration. Probe trials did not occur at any other place in the block, and only the probe trial could be out of order (50% of the time). In the Occluded conditions, this probe trial image was one of the instructed image set, rather than the occluder image. In the Visible condition, an image from the same set as in the preceding monitoring trials could be displayed out of order. As in the monitoring trials, participants respond(ed) to indicate whether the probe image was in or out of sequence.
- Feedback and reward: displayed (0.75 s) after the probe trial to indicate whether the participant’s response was correct (check mark) or not (X). The value of the condition was also displayed as $ (Low) or $$$ (High) on correct trials.

A single hyperblock was composed of two blocks of the same condition. The order of events in a hyperblock was as follows. The first block of a hyperblock began with a red screen that displayed the value. Participants pressed the assigned “in sequence” key to initiate the block (i.e., the block did not proceed without this response). After initiation, the red screen was followed by the four instruction images from a single image set, the monitoring trials (iterations 1–3), the probe trial during iteration 4, and then the feedback and reward. Between the first and second block of a hyperblock, a fixation cross was displayed for a variable ITI (0.25–8 s) before the green screen appeared. Participants pushed the “in sequence” button to start this second block, which proceeded immediately to the monitoring trials (iterations 1–3), the probe trial during iteration 4, and then the feedback and reward. A fixation cross was displayed during a variable ITI (0.25–8 s) before the start of the next hyperblock. For all blocks, if participants responded incorrectly on a monitoring trial before the probe trial (e.g., pressing the out of sequence key prematurely), the block was terminated: no further images and incorrect feedback (X) was immediately displayed.

#### Run structure

During scanning, participants completed six runs of the task. Each run lasted approximately 6 min and contained eight hyperblocks (16 total blocks). Each run included two hyperblocks of each of the four conditions (Occluded High, Occluded Low, Visible High, Visible Low). The two hyperblocks of each condition within each run used different image sets (e.g., Visible High set 1 and Visible High set 2; Fig. 1*D*). The order of the hyperblocks was randomized such that consecutive hyperblocks were not of the same condition and were counterbalanced across the six runs (Fig. 1*E*). In between runs, the scanner was stopped, and participants had the option of taking a brief break. Participants’ error rate (ER) on the previous run was displayed on the break screen to encourage correct performance.

#### Training

Participants were trained on the order of the four image sets immediately before scanning. During training (∼45 min), participants learned the reward value ($= Low, $$$ = High) and the correct ordering of the four images in each set by completing trials of the task. The meaning of the dollar signs was intentionally abstract to avoid participants explicitly “tallying” reward amounts across trials or blocks. Participants were instructed that they could earn a bonus of up to $10 based on their performance on a single run chosen at random, but they were not instructed a direct correspondence between dollar signs and bonus payment amount. We ensured that participants nevertheless understood and used the values associated with the sequences by training them before scanning and using a preference test described in further detail below.

To learn the correct order of the four image sets used for each participant, participants first completed the task without time constraints. The first time a new sequence was introduced, participants viewed the images serially without a response deadline, i.e., the image remained on the screen until a button press was made. Participants responded with the “j” and “k” keys on the keyboard to select a response with the index and middle finger of their right hand. Participants practiced two blocks with response times recorded, similar to the actual experiment. Training was conducted on the Visible condition of a sequence first, followed by practice of the Occluded version. Participants then completed two blocks of Occluded trials with response times recorded. This was repeated for each of the four sequences. Training was completed twice for each participant before moving on to completing the task in the scanner.

After training, participants completed a sequence preference test where they were instructed to select the high value sequence of each pair presented. Two of the four ordered image sets were displayed at a time: one set of four images across the top and one image set across the bottom of the screen relative to a central fixation spot. The image sets remained on the screen until one of the image sets was chosen with a button press (i.e., no response deadline), and there was a 1-s ITI between trials. All 24 combinations of image set pairs were displayed, and the location on the screen was counterbalanced across pairs and presentations. These trials were used to verify that participants had learned the associated reward value of the image sets.

After scanning, participants again completed the sequence preference test to verify that they had retained the learned image set reward values. A post-task questionnaire was administered to determine any factors that may have impacted performance and experience with the task during scanning (fatigue/sleep, sequence difficulty, response deadline, etc.), participants were debriefed and compensated for their time.

### Experimental design and statistical analysis

#### fMRI data acquisition and preprocessing

A Siemens 3T PRISMA MRI scanner with a 64-channel head coil was used for whole-brain imaging. Functional data were acquired using a fat saturated gradient-echo, echo planar imaging (EPI) pulse sequence (repetition time, TR = 2 s; echo time, TE = 28 ms; flip angle 90°; 38 interleaved axial slices; 3 × 3 × 3 mm). Anatomical scans included a T1-MPRAGE (TR, 1900 ms; TE, 3.02 ms; flip angle, 9°; 160 sagittal slices; 1 × 1 × 1 mm) and a T1 in-plane scan (TR, 350 ms; TE 2.5 ms; flip angle, 70°; 38 interleaved transversal slices; 1.5 × 1.5 × 3 mm).

Preprocessing and analysis were conducted in SPM12 (http://www.fil.ion.ucl.ac.uk/spm, RRID:SCR_007037). Two participants had one run of data removed because of excessive head motion (>3 mm). EPI images were slice time corrected and realigned to correct for head motion. Images were normalized to Montreal Neurologic Institute (MNI) stereotaxic space and smoothed with an 8-mm isotropic Gaussian kernel.

#### fMRI data analysis

Subject-specific models of condition effects were constructed in SPM12 under assumptions of the general linear model (GLM). Regressors were generated by convolving events of interest with the canonical hemodynamic response function and included the temporal derivative. If any trial in the block was incorrect or the participant stopped responding to the images, then the entire block was coded as an error because it was unknown whether the participant was correctly performing the monitoring trials. All models include a duration regressor based on the time the participant waited to start the trials at the block start screen (red or green) and an onset regressor modeled as a stick function for the feedback screen. Other conditions of interest are described below for each model. Nuisance regressors for all participants in all models included: instruction images, the first four monitoring trials in a block (iteration 1), error trials, the duration of the probe trial (including RT), a run regressor, and six motion parameters (translation and rotation).

For each participant, runs were entered as a single session and the first level was estimated as a fixed effects model. Whole-brain estimates of within subject effects were entered into second level random effects analyses. One-sample *t* tests were used to test for significance against zero (*p* < 0.001). Results were corrected for multiple comparisons based on whole-brain group effects with extent thresholds set at the cluster level, yielding a familywise error (FWE) correction (*p* < 0.05). Group contrasts are displayed on an inflated MNI canonical brain using Caret software (Van Essen et al., 2012; RRID:SCR_006260).

**Figure 2.**
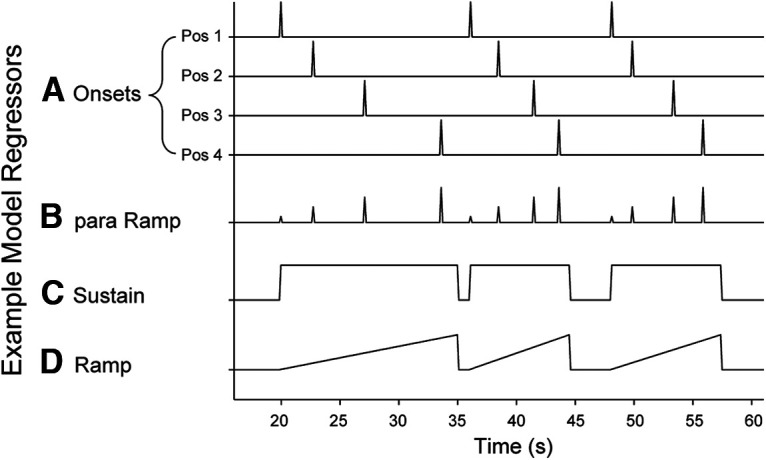
Example model regressors. ***A***, Onsets were modeled separately for each position as instantaneous (zero duration) events corresponding to the sequence position onsets model (Model 1; see Materials and Methods). ***B***, Parametric ramp regressors were used for the parametric sequence position (ramping) model. Linear increase across positions 1–4 with instantaneous onsets (Model 2). ***C***, Sustain regressors were constructed as a square wave from the onset of position 1 to the offset (response) of position 4 (models 3 and 4). ***D***, Ramp regressors linearly increased from the onset of the position 1 to the offset of position 4 (Models 3 and 4). All regressors shown were separated by visibility and reward conditions (e.g., a model containing onsets would contain 16 regressors: Occluded High positions 1–4; Occluded Low positions 1–4; Visible High positions 1–4; Visible Low positions 1–4). s = seconds. Figure adapted from Desrochers et al. (2015).

Five GLMs were estimated from the data as follows:

1. Sequence position onsets model. To assess the univariate effects of sequence position, cue visibility, and reward, we constructed a model using instantaneous stimulus onset regressors based on the factors of cue visibility (Occluded/Visible) × reward (High/Low) × sequence position (i.e., position within iteration, 1–4). We provide a simplified version of the regressors for the onsets in Figure 2*A*. All regressors shown were separated by visibility and reward conditions (e.g., a model containing onsets would contain 16 regressors: Occluded High positions 1–4; Occluded Low positions 1–4; Visible High positions 1–4; Visible Low positions 1–4).
2. Parametric sequence position (ramping) model. This ramping model tests for activation that increases with sequence position (Desrochers et al., 2015, 2019). Onset regressors were constructed by crossing the factors of cue visibility (Occluded/Visible) × reward (High/Low) to result in the four condition combinations. Sequence position (1–4) was added as a parametric modulator of the onsets for the positions. Temporal derivatives of the parametric regressors were also included. The parametric regressors are estimated hierarchically to account for variance above and beyond that explained by the onsets alone. Additionally, we included a separate nuisance regressor for the last image in a block (probe trial; Fig. 2*B*).
3. Sustain versus unique ramp model. We sought to identify whether variance in the fMRI signal was uniquely explained by sustained versus ramping activation. Models that included both sustained and ramping activation were constructed to allow the regressors to compete for variance within the same model. Sustain and ramp regressors (separate for each combination of cue visibility × reward) were included in addition to a single regressor for stimulus onset at each position. Sustain and ramp regressors started at stimulus onset of each sequence (position 1) and ended at the stimulus offset (button press) to sequence position 4. This initial model was used to identify variance uniquely explained by the ramp regressor. The sustain and ramp regressors were orthogonalized (spm_orth.m) within the condition combinations to remove shared variance from the ramp regressors (and assign it to the sustain regressors; Fig. 2*C*,*D*).
4. Unique sustain versus ramp model. This model complements the one above to identify variance uniquely explained by the sustain regressor (independent of ramp). The shared variance from the sustain regressors was removed and assigned to the ramp regressor. This model was used to identify variance uniquely explained by the sustain regressor. All other aspects of the model were the same as the previous sustain versus unique ramp model (Fig. 2*C*,*D*).
5. Block trial number onsets model. To determine how the fMRI signal evolves throughout the course of trials within a block, we modeled two complete sequence iterations (iterations 2 and 3) of each block as individual trials (excluding iteration 1 to avoid block initiation effects, see above, Block structure). Regressors were modeled as onsets as in Figure 2*A* but classified by trial across the block (5–12), for a total of eight regressors, instead of only four regressors for sequence position (1–4) as in Model 1.

a. Individual regressors were included for sequence positions (trials 5–12). Onset regressors were again included as nuisance regressors for the first four trials of the block to remove block initiation effects. For the remaining trials (13–16) in iteration 4 at the end of a block where the probe image could appear, an onset regressor of the same condition for each trial was included as a nuisance effect. All other aspects of the model were the same as Model 1. For the initial model, we collapsed across all conditions to examine overall block dynamics.
b. To examine monitoring without visible position cues, we created a model that included the same regressors above (5a) but included separate regressors for the Occluded and Visible conditions collapsed across reward conditions.
c. To examine reward influence, we created a model that included the same regressors as above (5a) but included separate regressors for the High and Low reward conditions collapsed across visibility conditions.
d. To examine the interaction between monitoring without visible position cues and reward, we created a model that included the same regressors above (5a) but included separate regressors for the combinations of Visible/Occluded and High/Low reward conditions.

#### Region of interest (ROI) analysis

ROIs were primarily defined from activation in the parametric ramp > baseline contrast in Desrochers et al. (2019). MNI coordinates for the RLPFC ROI defined by the cluster of activation in the parametric ramp > baseline contrast in Desrochers et al. (2019) are at *x* = −36, *y* = 42, *z* = 34 (center of mass) and will be hereafter referred to as the “D19” ROI. Limited *post hoc* supporting analyses were performed using the RLPFC cluster from the same contrast in the present study (across all conditions). The mean β values were extracted from the parametric ramp regressor across all voxels in the ROI using the Marsbar toolbox (MarsBar SPM toolbox, RRID:SCR_009605) in SPM. Ramping activation across models and regions were compared using repeated measures RMANOVA and paired *t* tests where appropriate. For the sequence position onsets and block trial number onsets models, the time course of activity across positions was extracted using an eight-timepoint (16 s) finite impulse response (FIR) model in the MarsBar toolbox in SPM that contained the same regressors as the onset model.

#### Behavioral analysis

As in previous studies (Desrochers et al., 2015, 2019), we excluded the first four trials of every block (iteration 1) from analysis to remove bias in RTs from block initiation effects (Schneider and Logan, 2006). RT analyses excluded error trials. To match data for examining trials across blocks, responses in the ITI (mean 8%, across participants), were included to avoid unnecessary data loss. For ER, we also conducted analyses to determine detection of an out of sequence item. We defined detection types as hits, correct rejections, misses, and false alarms to calculate d-prime. Hits were defined as correct responses to an out of sequence item. Correct rejections were correct responses to in sequence trials in both Visible and Occluded conditions. Misses were button presses indicating in sequence to an out of sequence item. False alarms were out of sequence responses to an in-sequence item. D-prime (*d’*) was calculated:

d′ =Z(hit rate)−Z(false alarm rate),

where Z(p),p∈[0,1], is the inverse of the normal cumulative distribution function (Macmillian and Creelman, 2004). Extreme rates of zero or one were converted to 1/(2*N*) and 1–1/(2*N*), with *N* being defined as the number of trials, to prevent an infinite d-prime (Macmillian and Creelman, 2004). RMANOVA and paired *t* tests were used to test for differences as described in Results. Analyses were conducted using MATLAB (MathWorks; RRID:SCR_001622).

## Results

### Behavioral results

To determine the effects of reward and cue visibility on sequence monitoring dynamics in the RLPFC, we used a sequence monitoring task [based on (Desrochers et al., 2019)] with value and visibility manipulations to create a two-by-two design (Fig. 1). Additionally, to separate processes related to sequence and reward, reward was provided after multiple sequences were performed (rather than one). Here, we use the term “iteration” to indicate one instance of the ordered image set, or one four-item sequence. In other words, rather than reward being associated with one iteration (as is typical), reward was provided after multiple sequence iterations, at the end of the block. During each block, participants monitored the sequential order of Visible or internally tracked (Occluded) items that were members of High or Low value sequences.

**Figure 3.**
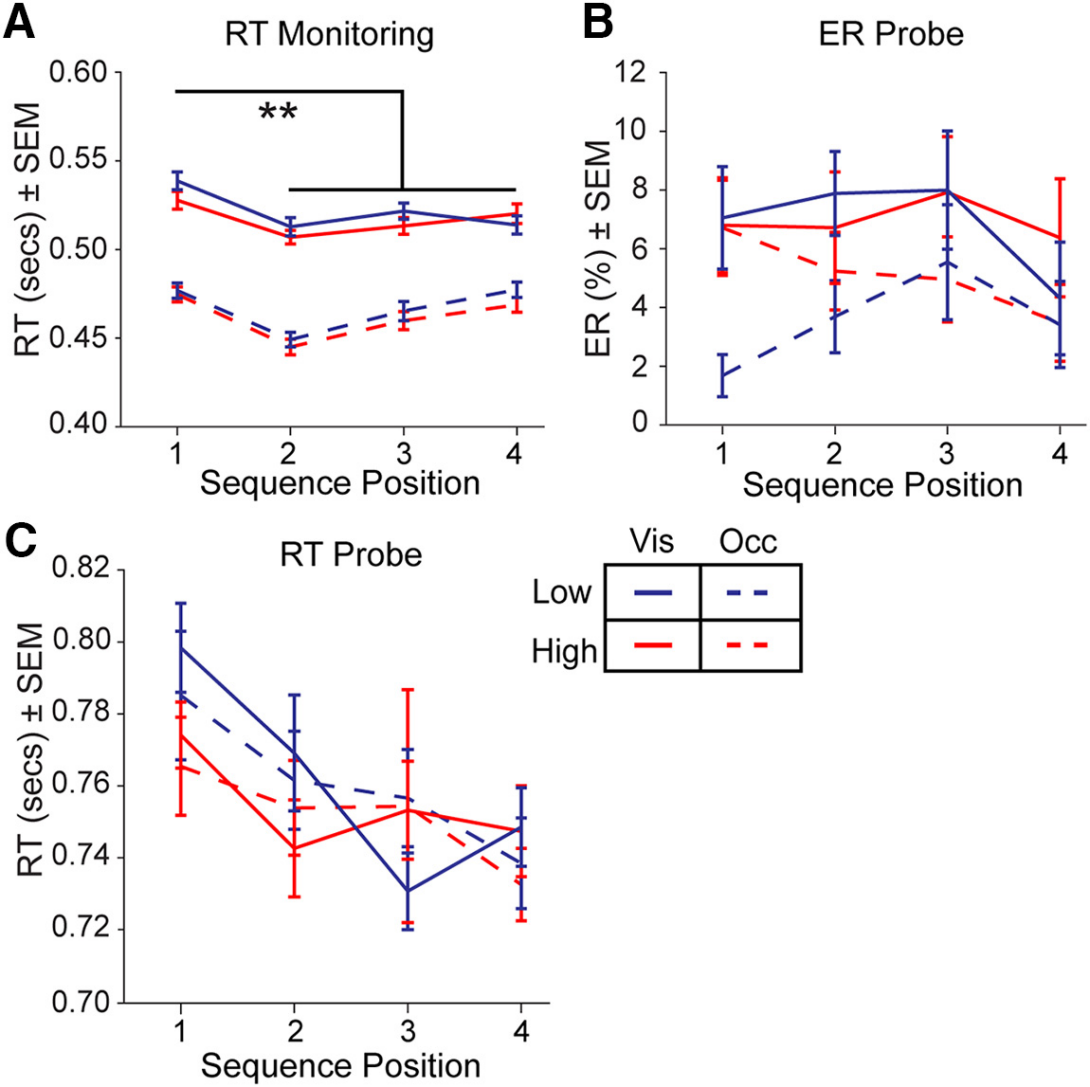
Behavioral results. ***A***, Mean RT across sequence position for monitoring trials. ***B***, Mean ER across sequence position for probe trials. ***C***, Mean RT across sequence position for probe trials. Solid lines depict the Visible condition and dashed lines depict the Occluded condition. Red color indicates High reward and blue color indicates Low reward. SEM = standard error of the mean. Vis = Visible condition. Occ = Occluded condition. **p < 0.001.

We first assessed RT on sequence monitoring trials (before the final probe trial) using a RMANOVA including factors for visibility (Visible/Occluded), reward (High/Low), and position [first position/subsequent positions (2–4)]. We replicated previous observations (Desrochers et al., 2015, 2019) that the RT at the first position in the sequence was slowed with respect to the subsequent positions in the sequence (2–4; RMANOVA, position: *F*_(1,35)_ = 25.7, *p* < 0.001; η_p_^2^ = 0.42; Fig. 3*A*). This sequence initiation cost provides evidence that participants were monitoring the items as sequences (Schneider and Logan, 2006). Participants performed the task well (mean ER, 12.5%), and as in previous studies there was no evidence of a sequence initiation cost in ER (RMANOVA, position: *F*_(1,35)_ = 0.02, *p* = 0.90, η_p_^2^ = 0.001; Fig. 3*B*).

Next, we assessed the effect of the cue visibility manipulation, Visible and Occluded conditions, on task performance. During monitoring, participants were faster on Occluded trials than Visible trials (RMANOVA, visibility: *F*_(1,35)_ = 83.9, *p* < 0.001, η_p_^2^ = 0.71; Fig. 3*A*). This RT difference could have resulted from the explicit cue (the occluder image) that indicated no further decision was necessary on Occluded monitoring trials. To test this prediction, we examined RT on probe trials, when cues were visible across both conditions. There were no reliable differences between the Visible and Occluded conditions on probe trials (RMANOVA, visibility: *F*_(1,35)_ = 0.12, *p* = 0.73, η_p_^2^ = 0.003), and we again replicated slower RT for the first relative to other positions in the sequence (RMANOVA, position: *F*_(1,35)_ = 15.3, *p* < 0.001, η_p_^2^ = 0.30; Fig. 3*C*). Errors during monitoring trials terminated the block of trials, and happened relatively infrequently (mean 0.73% of trials, across participants). Therefore, we examined ER on probe trials and found a greater ER for Visible compared with Occluded trials (RMANOVA, visibility: *F*_(1,35)_ = 5.2, *p* = 0.03, η_p_^2^ = 0.16). To further examine these differences in ER, we analyzed trials with respect to detecting an out of sequence item. There were no reliable differences in *d’* across conditions (RMANOVA: visibility, *F*_(1,35)_ = 3.3, *p* = 0.08, η_p_^2^ = 0.09; reward: *F*_(1,35)_ = 0.77, *p* = 0.39, η_p_^2^ = 0.02; interaction, *F*_(1,35)_ = 0.008, *p* = 0.93, η_p_^2^ = 0.0002). There were also no reliable differences in hits (RMANOVA: *F*s < 0.37, *p*s > 0.54) or false alarms (RMANOVA: *F*s < 1.01, *p*s > 0.32). In sum, although a difference existed in ER between Occluded and Visible conditions, this difference did not result in a reliable difference in the detection of out of sequence items.

To examine the effects of the reward manipulation on behavior, we compared High and Low value trials. Participants responded faster on High versus Low value sequences during monitoring trials (RMANOVA, reward: *F*_(1,35)_ = 4.9, *p* = 0.03, η_p_^2^ = 0.12) in a manner that did not interact with cue visibility (RMANOVA interaction: *F*_(1,35)_ = 0.60, *p* = 0.44, η_p_^2^ = 0.02; Fig. 3*A*). Note that while the effect of reward value on RT appears numerically small, the effect size could be interpreted as medium to large. This effect is consistent with a substantial literature illustrating faster RTs with increased reward (Niv, 2007; Guitart-Masip et al., 2011; Beierholm et al., 2013; Otto and Daw, 2019), and illustrates the efficacy of our reward manipulation. There was no effect of reward on probe trial ER (RMANOVA, reward: *F*_(1,35)_ = 3.6, *p* = 0.07, η_p_^2^ = 0.09) or any interaction between Occluded and Visible conditions (RMANOVA interaction: *F*_(1,35)_ = 1.7, *p* = 0.20, η_p_^2^ = 0.05; Fig. 3*B*). We also found no RT differences between reward conditions on probe trials (RMANOVA, reward: *F*_(1,35)_ = 2.7, *p* = 0.12, η_p_^2^ = 0.07) or interaction between reward and visibility conditions (RMANOVA, interaction: *F*_(1,35)_ = 0.02, *p* = 0.90, η_p_^2^= 0.0004; Fig. 3*C*). We confirmed that participants had learned and retained the values associated with the four image sets from the sequence preference test. Participants reliably selected the higher value image sets relative to chance during training (*t*_(35)_ = 45.4, *p* < 0.001) and after scanning (*t*_(35)_ = 54.1, *p* < 0.001). Further, there was no difference in preference between training and after scanning (*t*_(35)_ = 0.77, *p* = 0.45, *d* = 0.10).

### fMRI results

#### Effect of reward on individual sequences

**Figure 4.**
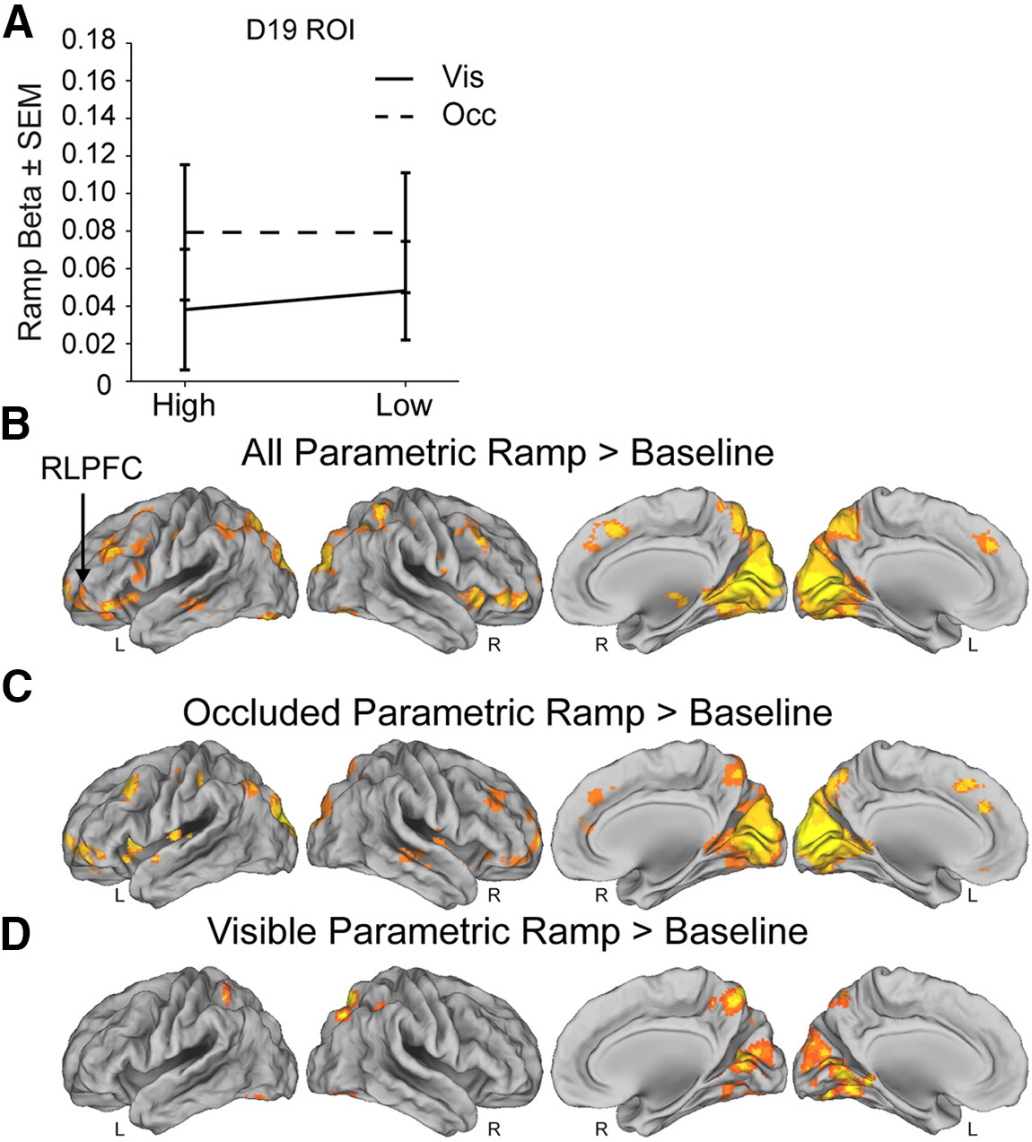
Ramping activity in ROI and whole-brain fMRI analyses. ***A***, Mean parametric ramp regressor β values for the D19 ROI in the parametric sequence position (ramping) model. Solid lines depict the Visible condition and dashed lines indicate the Occluded condition. ***B***, Ramping activation shown for the parametric ramp regressor over baseline contrast in the parametric sequence position (ramping) model (see Materials and Methods). FWE cluster corrected *p* = 0.05 (height *p* = 0.001, extent = 191 voxels). ***C***, Same model as in ***A*** but for the parametric ramp regressor for the Occluded condition over baseline contrast (FWE cluster corrected *p* = 0.05, height *p* =0.001, extent = 169 voxels). ***D***, Same model as in ***A***, ***B*** but for the parametric ramp regressor for the Visible condition over baseline contrast (FWE cluster corrected *p* = 0.05, height *p* = 0.001, extent = 180 voxels). Note that the direct contrast of parametric ramping in the Occluded and Visible conditions did not yield any clusters that survived statistical correction. SEM = standard error of the mean. Vis = Visible condition. Occ = Occluded condition.

To determine whether the task engaged ramping in the RLPFC as observed in previous studies (Desrochers et al., 2015, 2019), we defined a ROI from the parametric ramping cluster in RLPFC from Desrochers et al. (2019) during the sequence monitoring task (experiment 2) on which this task is based (“D19” ROI; see Materials and Methods). In the D19 ROI, we found that parametric ramping betas were significantly different from zero (*t* test: *t*_(25)_ = 3.0, *p* = 0.005, *d* = 0.5; Fig. 4*A*). Next, we examined whether variance in RLPFC could be better accounted for by ramping or sustained activation. We constructed a pair of models where ramp and sustain regressors competed for variance (see Materials and Methods) and then examined the variance in the MR signal in RLPFC that each regressor could uniquely account for (Fig. 2*C*,*D*). We found that variance in the D19 ROI was better accounted for by ramping, beyond what could be accounted for by sustained activation (*t* test: *t*_(35)_ = 6.1, *p* < 0.001, *d* = 2.5). These results were supported by a whole-brain voxelwise contrast of parametric ramping activity across all sequence conditions. We found ramping activation in the RLPFC that extended laterally into orbitofrontal cortex, as well as ramping in visual cortex, midcingulate, and SMA (Fig. 4*B*; Table 1). Thus, we replicated ramping activity during sequence monitoring with changes in reward value.

**Table 1:** Parametric ramp greater than baseline contrast activation values and brain areas

Location	Extent (voxels)	BA	*x*	*y*	*z*	Peak *t*-value
Insula	952	N/A	−38	31	−4	5.3
L RLPFC		10/11	−28	61	1	3.92
L inferior orbitofrontal cortex		47	−48	40	−8	5.21
L middle frontal gyrus		46	−30	52	28	4.59
L lateral orbitofrontal cortex		11	−28	64	4	4.36
L calcarine cortex	18,091	17	−8	−80	14	8.42
R calcarine cortex		17	14	−66	8	7.44
L lingual gyrus		18	−8	−58	−4	6.50
R lingual gyrus		18	20	−76	−16	6.37
L cuneus		19	−6	−84	42	6.44
L superior parietal lobule		7	−4	−62	60	6.29
L supramarginal gyrus		40	−40	−52	54	5.51
L supramarginal gyrus		2	−46	−28	48	3.48
R supramarginal gyrus		40	42	−38	40	5.03
R superior occipital gyrus		19	18	−72	42	5.26
R middle occipital gyrus		19	34	−76	22	3.52
L fusiform gyrus		37	−44	−62	−24	4.82
R fusiform gyrus		37	42	−50	−28	4.35
L cerebellum		N/A	−24	−76	−18	6.93
R superior frontal gyrus	1898	9	38	32	44	5.23
R SMA		6	26	6	62	5.12
R postcentral gyrus		3	40	−12	40	4.63
IFG, Triangularis		48	48	16	22	4.14
L thalamus		N/A	−12	−18	−4	5.04
R inferior frontal (orbital)	953	47	36	28	−8	5.42
R lateral orbitofrontal cortex		11	24	66	2	5.02
R middle frontal gyrus		46	42	52	12	3.72
R middle frontal gyrus		46	38	50	24	3.59
R middle cingulate	735	32	2	42	34	5.49
L middle frontal gyrus	657	44	−48	24	38	6.04
L SMA		6	−30	4	38	4.09
R middle temporal gyrus	343	21	46	−34	−8	4.4
L Central Operculum	309	48	−42	−14	32	5
L IFG, opercularis		48	−60	−4	14	3.94
L inferior temporal gyrus	207	20	−40	−6	−16	5.73

All peaks are greater than 25 mm apart (cluster corrected *p* = 0.05 FWE). Extent is the cluster size in voxels, listed for each peak belonging to the same cluster. BA = Brodmann’s area. *x*, *y*, *z* are MNI coordinates. IFG, inferior frontal gyrus; SMA, supplementary motor area. N/A = not applicable.

To determine whether monitoring without visible sequence position cues influenced RLPFC ramping in the context of changes in reward, we next examined ramping activity in the Visible and Occluded conditions. These analyses were performed in the parametric ramping model that contained all four (two-by-two) conditions. In the D19 ROI, although the parametric ramp regressor betas were numerically greater in the Occluded than the Visible condition (Visible: Mean = 0.04, Occluded: Mean = 0.08), there was no reliable difference between them (RMANOVA, visibility: *F*_(1,35)_ = 1.2, *p* = 0.28, η_p_^2^ = 0.03; Fig. 4*A*). Whole-brain voxelwise contrasts of these conditions supported these results. Although there were a greater number of areas, including the RLPFC, that survived statistical correction in the Occluded condition (Fig. 4*C*; Table 2) compared with the visible condition (Fig. 4*D*; Table 3), no clusters survived statistical correction in the direct contrast of these conditions. A follow-up test using an ROI defined by the RLPFC cluster in the parametric ramping contrast across conditions in the current experiment (Fig. 4*B*; Table 1) also did not show a reliable difference between ramping betas in Occluded and Visible conditions (RMANOVA, visibility: *F*_(1,35)_ = 0.98, *p* = 0.33, η_p_^2^ = 0.03). These results replicate previous findings (Desrochers et al., 2019) and suggest that memory processes that are not explicitly sequential do not interact with reward-related dynamics in this task. However, these results do not rule out the possibility, and we directly test these potential interactions between reward and memory in the context of sequence monitoring dynamics in the RLPFC below.

**Table 2:** Occluded condition parametric ramp greater than baseline contrast activation values and brain areas

Location	Extent (voxels)	BA	*x*	*y*	*z*	Peak *t*-value
R calcarine cortex	11,971	17	10	−78	4	9.76
L calcarine cortex		17	−18	−70	8	6.9
L calcarine cortex		17	−2	−92	2	7.16
R superior parietal lobule		7	4	−64	52	5.63
R superior parietal lobule		7	32	−66	54	4.82
L fusiform gyrus		37	−26	−64	−8	3.67
R fusiform gyrus		37	26	−60	−14	4.8
R lingual gyrus		18	8	−48	2	4.59
R ventral posterior cingulate		23	20	−58	28	4.19
R inferior frontal (orbital)	1251	47	40	28	−6	4.91
R middle temporal gyrus		21	58	−28	−8	4.81
R IFG, opercularis		48	62	−4	8	4.65
L middle temporal gyrus	1046	21	−48	−4	−12	5.7
L superior temporal		48	−48	−24	10	4.45
R IFG, opercularis		48	−40	20	2	4.98
R anterior cingulate	703	32	2	38	30	5.23
L anterior cingulate		32	−14	16	46	3.6
R ventromedial prefrontal cortex		11	10	40	2	3.67
R RLPFC	398	10	18	66	6	5.68
R middle frontal orbital gyrus		47	44	48	−14	4.54
L ventromedial prefrontal cortex	558	11	−12	34	−16	5.23
L RLPFC		10	−22	64	4	5.16
L inferior frontal (orbital)		47	−42	52	−12	4.76
R middle frontal gyrus	376	9	30	36	38	4.71
L supramarginal gyrus	340	2	−42	−28	48	4.52
L middle frontal gyrus	303	46	−38	22	42	4.56
L angular gyrus	227	39	−40	−58	50	4.05

All peaks are greater than 25 mm apart (cluster corrected *p* = 0.05 FWE). Extent is the cluster size in voxels, listed for each peak belonging to the same cluster. BA = Brodmann’s area. *x*, *y*, *z* are MNI coordinates. IFG, inferior frontal gyrus.

**Table 3:** Visible condition parametric ramp greater than baseline contrast activation values and brain areas

Location	Extent (voxels)	BA	*x*	*y*	*z*	Peak *t*-value
R superior parietal lobule	6746	7	14	−68	58	5.86
L calcarine cortex		17	−12	−80	12	5.77
R calcarine cortex		17	12	−64	14	4.98
R calcarine cortex		17	12	−64	14	4.55
L lingual gyrus		18	−12	−58	−12	5.61
L superior occipital gyrus		19	−8	−82	46	4.13
R superior occipital gyrus		18	14	−90	26	3.77
L fusiform gyrus		37	−34	−74	−16	4.55
R fusiform gyrus		37	32	−76	−12	4.37
R supramarginal gyrus		40	42	−36	44	4.75
R fusiform gyrus		37	28	−48	−8	3.55
L supramarginal gyrus	528	40	−40	−50	52	5.62

All peaks are greater than 25 mm apart (cluster corrected *p* = 0.05 FWE). Extent is the cluster size in voxels, listed for each peak belonging to the same cluster. BA = Brodmann’s area; *x*, *y*, *z* are MNI coordinates.

To test our central hypothesis that changes in reward will cause changes in individual sequence RLPFC ramping dynamics, we examined the High and Low reward conditions in the same parametric ramping model. In the D19 ROI, there was no difference between parametric ramp regressor betas in the High and Low reward conditions (RMANOVA, reward: *F*_(1,35)_ = 0.01, *p* = 0.91, η_p_^2^ = 0.002; Fig. 4*A*). A follow-up whole-brain contrast of the parametric ramping activity in the High and Low reward conditions revealed no clusters that survived statistical correction. However, this result does not exclude the possibility of an interaction such that reward may only exert an influence on individual sequence ramping dynamics in RLPFC under particular visibility conditions. Interactions between memory and other cognitive control processes have been observed in lateral prefrontal cortex (Jimura et al., 2010). In the D19 ROI there was no interaction between parametric ramp regressor β values between the cue visibility and reward conditions (RMANOVA: *F*s < 1.21, *p*s > 0.28; Fig. 4*A*). These results were also consistent with results in the RLPFC ROI defined from the current experiment parametric ramping contrast, and did not show a significant difference between ramping betas in High versus Low conditions (RMANOVA, reward: *F*_(1,35)_ = 0.08, *p* = 0.78, *d* = 0.001) or interaction between cue visibility and reward conditions (RMANOVA interaction: *F*_(1,35)_ =1.5, *p* = 0.23, η_p_^2^ = 0.04). Thus, these results did not support the hypothesis that increased reward would result in an overall increase in magnitude or change in slope in the ramping activity in the RLPFC for individual sequences (iterations). The influence of reward on sequence monitoring processes may be more complex than greater activity potentially indicative of a generally heightened state of engagement. We therefore next tested whether reward causes changes in RLPFC sequence tracking dynamics that change across sequence iterations.

#### Effect of reward across sequence iterations

**Figure 5.**
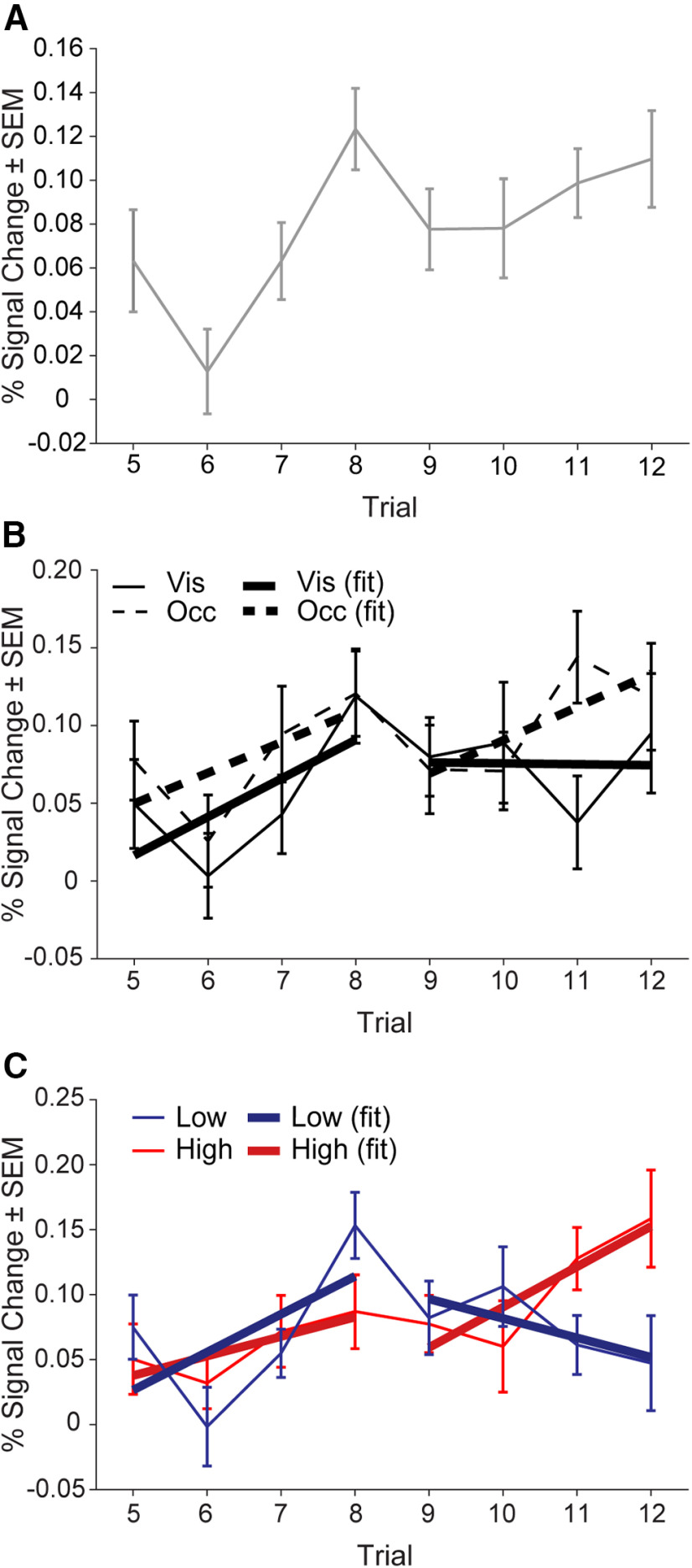
Activity in the D19 ROI changes across a block of trials. ***A***, Block trial number onsets model collapsed across all conditions. ***B***, Block trial number onsets model by visibility conditions, collapsed across reward conditions. Solid lines depict the Visible condition and dashed lines indicate the Occluded condition. Actual data are shown with error bars and SEM, standard error of the mean. Line fit based on sequence iteration is shown in thicker lines of the same type (solid/dashed). ***C***, Block trial number onsets model by reward condition, collapsed across visibility conditions. Red color indicates High reward and blue color indicates Low reward. Actual data are shown with error bars and SEM. Line fit based on sequence iteration is shown in thick and darkened lines of the same color (red/blue). Mean percent signal change (%) (±SEM) from the peak (2–4 s) of the FIR.

To test for changes across sequence iterations as a result of reward, we first examined RLPFC dynamics on a longer time scale than single sequences, i.e., across blocks of trials. Collapsing across conditions, we modeled the two complete sequence iterations of each block (iterations 2 and 3, trials 5–12) as individual trials to quantify activity in the RLPFC across the block. The first iteration of the block (trials 1–4) was excluded to avoid block initiation effects (model 5a; see Materials and Methods). In the D19 ROI, RLPFC activity appeared to increase for each sequence iteration and through the block overall (RMANOVA linear contrast: *F*_(1,35)_ = 7.3, *p* = 0.01, η_p_^2^ = 0.17; Fig. 5*A*). To test whether ramping activity “resets” for each sequence iteration independent of the overall increase, we detrended the activity across the block by fitting a simple linear regression across all trials 5–12 and examining the resulting residuals. Because we hypothesized that this “reset” occurs at position 1 of each sequence iteration, we tested for differences in the first positions after detrending. In the D19 ROI, there was no difference between the first position trials in the residuals (trial 5 vs trial 9; *t* test: *t*_(35)_ = 0.68, *p* = 0.50, *d* = 0.15). Importantly, these results provide evidence that sequence specific dynamics are separable from more generalized block dynamics in the RLPFC (see also Wen et al., 2020) and provide a foundation for further examining the potential influence of memory and reward across sequence iterations.

To examine the effects of monitoring sequences without visible position cues on across-block dynamics in the RLPFC, we created a model that again included individual regressors for trials 5–12 and separate regressors for Occluded and visible conditions (collapsed across reward conditions, model 5b; see Materials and Methods). In the D19 ROI activity, there was no interaction between trial number and visibility condition (RMANOVA interaction: *F*_(7,245)_ = 0.75, *p* = 0.63, η_p_^2^ = 0.02; Fig. 5*B*). In addition, we observed a small but reliable difference between Occluded and Visible activity across the block (RMANOVA, visibility: *F*_(1,35)_ = 4.3, *p* = 0.047, η_p_^2^ = 0.11; Fig. 5*B*). This greater RLPFC activation in Occluded trials across the block contrasts with results presented above when examining ramping β values collapsed across sequence iteration. These results suggest that there is an overall increase in RLPFC activity in Occluded compared with Visible trials that does not change the slope of the ramping activation.

To address the central question of whether ramping activity in the RLPFC is affected by reward differently across sequence iterations, we used the same block trial number onsets model (iterations 2 and 3, trials 5–12) and included separate regressors for High and Low reward conditions (across visibility conditions, model 5c; see Materials and Methods). Across the block, we found that the difference in activity in the D19 ROI for the High and Low reward conditions changed across trials such that there was a greater difference as the ending reward approached (RMANOVA interaction: *F*_(1,35)_ = 2.3, *p* = 0.02, η_p_^2^ = 0.06; Fig. 5*C*). To further examine these dynamics, we compared the slope of the lines fit to the activity for each condition in the first and second sequence iteration separately. Interestingly, we found that for the first iteration of the sequence, ramping activity in the D19 ROI for High and Low reward conditions was very similar (trials 5–8; ANCOVA: *F*_(1,284)_ = 0.65, *p* = 0.42, η_p_^2^ = 0.002). In contrast, ramping activity had significantly greater slope for the High compared with the Low reward condition in the second sequence iteration (trials 9–12; ANCOVA: *F*_(1,284)_ = 4.8, *p* = 0.03, η_p_^2^ = 0.02). Together, these results suggest that reward information, although it influences behavior throughout the block, is reflected in RLPFC activity only as the end of the block approaches. Further, these results imply that reward information is integrated into temporally extended control processes closer to points in time when the information is most relevant.

We also performed an exploratory analysis to determine whether these across-block reward effects in the RLPFC changed when participants monitored the sequences with or without visual cues. For this block trial number onsets model, we constructed separate regressors for trials 5–12, Occluded, Visible, High and Low reward conditions (model 5d; see Materials and Methods). In the D19 ROI, we found no reliable interactions between reward and cue visibility conditions (RMANOVA: *F*s < 1.13, *p*s > 0.34). Although we cannot rule out the possibility that memory and reward processes may interact during sequence monitoring, these data do not provide evidence that such an interaction occurs in the RLPFC.

## Discussion

In this experiment, we examined the effects of reward on ramping dynamics in the RLPFC across the monitoring of multiple sequence iterations. We tested three possibilities for how changes in RLPFC dynamics might emerge: through an interaction with a memory process, as a consistent change in all individual sequences, and as a change across sequence iterations. We provide novel evidence that reward does not affect sequence tracking dynamics in the RLPFC as either a tonic increase or a change in slope consistently across individual sequences. Further, these effects do not interact with whether sequential items are monitored with visible cues. In contrast, we observed effects of reward that changed across sequence iterations, with a rapid increase in amplitude with proximity to high reward. In other brain areas and in recent computational models (Kim et al., 2020; Ott et al., 2020; Cruz and Paton, 2021; Hamid et al., 2021) this “accelerating” impact is a hallmark of reward signaling in specific forebrain representations that are responsible for task completion, and possess the correct predictive model of ongoing events. This finding adds to prior imaging, TMS and lesion studies all indicating a distinctive role for RLPFC in sequence processing.

Similarities between blood oxygen level-dependent (BOLD) activity in the RLPFC and dopamine dynamics observed in the striatum of rodents performing temporally extended tasks (Howe et al., 2013; Hamid et al., 2016, 2021) has intriguing implications for RLPFC function. Dopamine dynamics that bifurcate only in the latter half of a sequential task between high and low rewards have been observed in the striatum of rats (Howe et al., 2013). Dopamine concentration that accelerates to reward in such studies has also been used as evidence in support of a single motivational signal for adapting to current and future behavior (Hamid et al., 2016), in contrast to separate tonic and phasic dopamine signaling (Cohen et al., 2002). Further, in subsequent studies, such dopamine signaling was only observed in situations where reward was contingent on action (i.e., instrumental) rather than not (i.e., Pavlovian), and only in brain areas that were involved in the instrumental actions (Hamid et al., 2021). Companion computational models suggest these dynamics drive credit assignment specific to involved brain regions. Together, these studies suggest that the RLPFC is performing an active monitoring function that integrates reward and sequence information, rather than a passive observer of signals relayed from elsewhere in the brain.

An interesting qualitative observation from our data are that we found ramping dynamics specific to individual sequences and that extended throughout task blocks in RLPFC. This finding further reinforces the idea that RLPFC simultaneously processes not only local sequences, but the multiscale sequences that are inherent in complex behavior. For example, when cooking, it is necessary not only to track the sequence of steps in what you are currently cutting up, but also to track where in the overall sequence of meal preparation steps that cutting exists. These dynamics parallel recent findings from Wen et al. (2020), who showed that the RLPFC and other “multiple demand” regions were sensitive to step-level and episode-level information and preferentially represented step (rather than task) identity. We extend these results by illustrating that reward information is also incorporated into signals in the RLPFC. Further studies are necessary to determine the mechanisms by which these control signals are multiplexed during sequences.

Our results are consistent with an integration account of RLPFC function. In nonsequential tasks, RLPFC has been shown to integrate multiple sources of task information, such as different stimulus dimensions (Nee et al., 2014), mental arithmetic operations (De Pisapia et al., 2007, 2012), relational integration in the visuospatial (Christoff et al., 2001) or semantic domain (Bunge et al., 2005), and motivation during task updating (Bahlmann et al., 2015). These cognitive control paradigms required that context and task goals be flexibly updated during specific temporal windows dependent on task demands. We extend these findings to sequential cognitive control by demonstrating that ramping dynamics accelerate based on temporal context, such as proximity to end reward. In this context, our results suggest that RLPFC is uniquely suited during temporally extended behaviors to integrate reward information dependent on task relevance.

There were limitations to the present study. First, we focused on the RLPFC because, although its general role in sequential processes has been established, the specifics of how its dynamics interact with myriad other variables key to sequential control has only begun to be investigated. The RLPFC is also part of a network of areas that display ramping dynamics (Fig. 4), and while an investigation of all these areas is outside the scope of the current experiment, it remains an important avenue of future research. Second, while the memory manipulation applied here showed no unique interactions and addressed potential differences in monitoring with and without external position cues, there remains the possibility that other memory processes may interact with reward (Wimmer et al., 2014; Shigemune et al., 2017) that were not explicitly tested in this experiment. For example, with visible position cues, there is likely a retrieval process that is still necessary to “check” that the cue is in the correct, remembered position. Third, we manipulated a single type of reward. Primary reinforcers such as foods, intrinsic rewards, and the valence of the reward (e.g., avoiding punishment) have potentially differing effects (Braver et al., 2014). Fourth, the failure to find interactions between reward value and the visibility of cues across blocks of trials should be interpreted with caution, as it is possible that we were not sufficiently powered to detect such interactions. The present work establishes a foundation on which to test these and other related variables.

In summary, this study suggests that sequential control dynamics in the RLPFC reflect an accelerating, dopamine-related reward signal in addition to both local and more extended position information. Understanding how rewards are integrated into more complex and extended timescale sequential decision processes has important implications for understanding human behavior in health and in disorders such as addiction. The multiscale nature of these signals in the RLPFC and their interaction with dopaminergic neurons in other regions that display similar ramping dynamics will be important to examine in the future, both in human and nonhuman primate models.

## Synthesis

Reviewing Editor: Ifat Levy, Yale University School of Medicine Decisions are customarily a result of the Reviewing Editor and the peer reviewers coming together and discussing their recommendations until a consensus is reached. When revisions are invited, a fact-based synthesis statement explaining their decision and outlining what is needed to prepare a revision will be listed below. The following reviewer(s) agreed to reveal their identity: NONE. Note: If this manuscript was transferred from JNeurosci and a decision was made to accept the manuscript without peer review, a brief statement to this effect will instead be what is listed below.

The authors have done a good job revising the manuscript.
